# Valproic Acid Increases CD133 Positive Cells that Show Low Sensitivity to Cytostatics in Neuroblastoma

**DOI:** 10.1371/journal.pone.0162916

**Published:** 2016-09-14

**Authors:** Mohamed Ashraf Khalil, Jan Hraběta, Tomáš Groh, Pavel Procházka, Helena Doktorová, Tomáš Eckschlager

**Affiliations:** 1 Department of Pediatric Hematology and Oncology, 2nd Medical Faculty, Charles University and University Hospital Motol, Prague, Czech Republic; 2 Department of Biochemistry, Faculty of Science, Prague, Czech Republic; 3 Institute of Experimental Medicine, Academy of Sciences, Prague, Czech Republic; 4 Institute of Biology and Medical Genetics, 1st Medical Faculty, Charles University, Prague, Czech Republic; Second University of Naples, ITALY

## Abstract

Valproic acid (VPA) is a well-known antiepileptic drug that exhibits antitumor activities through its action as a histone deacetylase inhibitor. CD133 is considered to be a cancer stem cell marker in several tumors including neuroblastoma. CD133 transcription is strictly regulated by epigenetic modifications. We evaluated the epigenetic effects of treatment with 1mM VPA and its influence on the expression of CD133 in four human neuroblastoma cell lines. Chemoresistance and cell cycle of CD133+ and CD133− populations were examined by flow cytometry. We performed bisulfite conversion followed by methylation-sensitive high resolution melting analysis to assess the methylation status of CD133 promoters P1 and P3. Our results revealed that VPA induced CD133 expression that was associated with increased acetylation of histones H3 and H4. On treatment with VPA and cytostatics, CD133+ cells were mainly detected in the S and G2/M phases of the cell cycle and they showed less activated caspase-3 compared to CD133− cells. UKF-NB-3 neuroblastoma cells which express CD133 displayed higher colony and neurosphere formation capacities when treated with VPA, unlike IMR-32 which lacks for CD133 protein. Induction of CD133 in UKF-NB-3 was associated with increased expression of phosphorylated Akt and pluripotency transcription factors Nanog, Oct-4 and Sox2. VPA did not induce CD133 expression in cell lines with methylated P1 and P3 promoters, where the CD133 protein was not detected. Applying the demethylating agent 5-aza-2’-deoxycytidine to the cell lines with methylated promoters resulted in CD133 re-expression that was associated with a drop in P1 and P3 methylation level. In conclusion, CD133 expression in neuroblastoma can be regulated by histone acetylation and/or methylation of its CpG promoters. VPA can induce CD133+ cells which display high proliferation potential and low sensitivity to cytostatics in neuroblastoma. These results give new insight into the possible limitations to use VPA in cancer therapy.

## Introduction

Valproic acid (VPA) is a widely used drug in the treatment of epilepsy and other neurological disorders. Recently, it belongs to a group of anticancer agents known as histone deacetylase (HDAC) inhibitors. HDAC inhibitors promote the histone acetylation in the nucleosomal structure, thereby keeping the chromatin in a relaxed form with consequent activation of many genomic regions [1]. HDAC inhibitors are promising anticancer drugs because they can restore the balance between histone acetylation and deacetylation which is often disturbed in cancer, resulting in chromatin remodeling which may enhance the recovery of multiple silenced antitumor genes [2]. The mechanism of VPA as a HDAC inhibitor acts through inhibition of HDACs class I and IIa that will differentially activate a wide range of nuclear and cytoplasmic proteins depending on tumor cell biology [3]. VPA does not only suppress tumor growth and induce apoptosis in cancer cells, but it also has anti-angiogenic effects and can induce tumor differentiation [4]. A number of HDAC inhibitors including VPA are currently under evaluation in clinical trials while vorinostat, romidepsin and belinostat have already been registered for treatment of some types of T-cell lymphomas [5]. However, the exact anticancer mechanism of VPA is still unclear and it exhibits different effects in various tumors [4]. For instance, VPA has shown to inhibit the invasiveness in bladder cancer but not in prostate cancer cells [6] and it did not induce cell cycle inhibition in some neuroblastoma cell lines such as SH-SY5Y and SK-N-BE [7]. Moreover, the expression of the pluripotency factor *Fgf4* decreased in F9 embryonal carcinoma cell line after treatment with VPA while elevated in P19 cells [8]. Collectively, these remarks lead to suggest that the anticancer effect of VPA may be cancer type specific and dose dependent [9]. On the other hand, the growing assumption about the role of HDAC inhibitors as potential candidates for inducing the pluripotent stem cells has been confirmed in some studies [10]. For example, the significant effect of VPA on amplification and maintenance of human hematopoietic stem cells [11,12], enhancement of the epithelial mesenchymal transition of colorectal cancer cells [13] and induction of CD133 in human glioma [14] have been reported in different studies. These previous results raise a question whether treatment with VPA may amplify cancer cells with stem cell features such as CD133+ cells.

Cancer stem cell (CSC) model presumes that tumor is not a homogenous population but contains a subpopulation of tumor initiating cells known as CSCs. This theory suggests that CSCs rather than the majority of tumor cells are responsible for driving tumor growth and progression [15]. CD133+ cells were identified as CSCs in NB and other brain tumors [16]. According to CSC theory, targeting CSCs may lead to tumor differentiation and degeneration. Therefore, CD133+ cells should be taken as a target in modern cancer therapy.

CD133, also known as Prominin-1, is a transmembrane glycoprotein that is expressed on neural precursor cells of postnatal cerebellum [17] and several brain tumor cells [18]. It has been reported that CD133 transcription is controlled by epigenetic modifications such as histone acetylation and promoter methylation [19–21]. Thus, it is supposed that this correlation between CD133 expression and acetylation status of histones enables the HDAC inhibitors to act as a potent regulator of CD133 transcription. In addition, CD133 promoters P1, P2 and P3 have high CpG content therefore they may undergo DNA methylation with consequent repression of gene transcription [22].

CD133+ cells display CSCs features such as self-renewal capacity [23], high clonogenicity [24], high proliferation potential *in vitro* [25] and formation of neurospheres in serum free medium [26]. Several groups have also shown that sorted CD133+ cells displayed greater tumorigenicity and resistance to chemotherapeutic agents compared to CD133− cells [27–31]. Furthermore, Wei Y *et al*., have recently clarified the functional association of CD133 molecule in the activation of PI3K/Akt pathway that promotes the tumorigenic capacity in glioma stem cells [32]. PI3K/Akt pathway is considered to be one of the most potent pro-survival signaling pathways that is activated in many types of cancer and associated with poor outcome in neuroblastoma [33].

Neuroblastoma (NB) is an extracranial pediatric solid tumor of neuroectodermal origin. NBs are biologically heterogeneous; the low-risk form may regress or mature spontaneously while the high-risk form grows relentlessly and can be rapidly fatal. Prognosis of high-risk NB tumors is still poor because drug resistance arises in the majority of patients. Some NB cells can retain multipotency and highly express stem cell related genes such as Oct-4 [34]. Thus, the role of CSCs in NB growth and progression is being widely accepted [16]. In addition, high expression of CD133 has been linked to a poor prognosis in several tumors including NB [35–39]. Based on previous studies, we were interested to evaluate the effect of VPA treatment on expression of CD133+ cells in NB derived cell lines.

## Materials and Methods

### Cell lines and chemicals

Human NB cell lines UKF-NB-3 and UKF-NB-4 [40], established from bone marrow metastases of high-risk NB, were a gift from Prof. J. Cinatl, Jr. (J. W. Goethe University, Frankfurt, Germany). IMR-32 and SH-SY5Y were purchased from ECACC, Salisbury, UK. Cells were cultivated in Iscove’s modified Dulbecco’s medium (IMDM) supplemented with 10% fetal bovine serum (FBS) and 1% penicillin/streptomycin (all from Gibco Life Technologies, Carlsbad, CA, USA). Cultured cells were grown in a humid incubator at 37°C and 5% CO_2_. Sodium salt of valproic acid (VPA), trichostatin-A (TSA), vorinostate (SAHA), entinostat (MS-275) and valpromide (VPM) were purchased from Sigma Chemical Co. (St. Louis, MO, USA). VPA was dissolved in IMDM while other HDAC inhibitors (TSA, SAHA and MS-275) and VPA analogue (VPM) were dissolved in DMSO that was used in final concentration not exceeding 0.3%. Vincristine sulfate (VCR) and cisplatin (CDDP) were obtained from Teva Pharmaceuticals (Prague, Czech Republic) and PLIVA-Lachema (Brno, Czech Republic) respectively.

### Clonogenic and neurosphere assay

For performing clonogenic assay, cells pretreated with 1mM VPA for 72 hrs and the untreated control were counted using TC20 cell counter (Bio-Rad Laboratories, Hercules, CA, USA). Hundred cells per well were seeded in 6-well plates (TPP Techno Plastic Products AG, Trasadingen, Switzerland) and left to grow in IMDM with 10% FBS at 37°C and 5% CO_2_ for 14 days. Colonies were fixed in acetic acid/methanol 1:7 solution for 5 min, then the fixative solution was withdrawn and colonies were left to dry. Dried colonies were stained with Giemsa overnight. Colonies greater than 50 cells were counted and plating efficiency was calculated as follows: plating efficiency = (number of colonies formed / number of cells seeded) × 100.

For neurosphere assay, cells were seeded at a density of 100 cells per well in 6-well ultra-low attachment multiwall polystyrene plates (Falcon, Becton Dickinson, Franklin Lakes, NJ, USA). Cells were maintained in serum-free media (SFM) contained a 1:1 mixture of Ham´s F12 Nutrient Mixture and advanced DMEM (both from Gibco Life Technologies), supplemented with 40 ng/ml EGF, 20ng/ml bFGF (both from Invitrogen, Fisher Scientific), 1% B27 supplement (Gibco Life Technologies), 2 μg/ml heparin (Sigma Aldrich) and 100 units/ml penicillin/streptomycin. Aliquots of EGF and bFGF were supplied every other day. On day 14, formed neurospheres were counted under microscope (Olympus IX51; Olympus Corporation, Tokyo, Japan).

### Western blot

All cells were collected and lysed in RIPA buffer containing protease inhibitors (Complete, Roche Diagnostics, Basel, Switzerland). Protein concentration was measured using DC protein assay (Bio-Rad Laboratories, Hercules, CA, USA) according to the manufacturer’s instructions. Thirty micrograms of extracted proteins were separated by SDS-PAGE electrophoresis using 10% gel. Separated proteins were transferred to a nitrocellulose membrane and blocked with 3% non-fat milk for one hour at 4°C. The membrane was exposed to mouse anti-human CD133/1 clone W6B3C1 (1:100; Miltenyi Biotec GmbH, Bergisch Gladbach, Germany) as the primary antibody for one hour at room temperature. Membrane was washed and exposed to peroxidase conjugated anti-mouse IgG secondary antibody (1:2000; Bio-Rad Laboratories) for 10 minutes. Antigen-antibody complex was visualized using an enhanced Immun-Star HRP Substrate chemiluminescence detection system (Bio-Rad Laboratories) according to the manufacturer’s instructions. Beta-actin antibody (1:2000; Sigma-Aldrich) was used as a loading control. For detection of phosphorylated Akt (P-Akt), we used rabbit monoclonal anti-P-Akt (Ser473) antibody (clone D9E) and rabbit polyclonal anti-Akt (total) antibody (both, 1:1000; Cell Signaling Technology Inc., Beverly, MA, USA). Pluripotency transcriptional factors were examined according to the manufacturer’s instructions using StemLight™ Pluripotency Transcription Factor Antibody Kit (Cell Signaling Technology) containing rabbit monoclonal anti Oct-4A (clone C30A3), Sox2 (clone D609) and Nanog (clone D73G4) antibodies. Peroxidase conjugated anti-rabbit IgG (1:2000; Bio-Rad Laboratories) was used as secondary antibody.

To estimate the acetylation of histones H3 and H4, isolation of histones was performed as described by Shechter et al., through acid extraction of histones followed by precipitation using trichloracetic acid [41]. Western blot was performed as mentioned above using 2 μg of isolated histones and separated on 16% gel. The membrane was exposed to rabbit polyclonal anti-acetyl-histone H3 (1:4000) and anti-acetyl-histone H4 (1:1000) primary antibodies (both, Upstate Biotechnology Inc., Lake Placid, NY, USA) overnight at 4°C. Peroxidase conjugated anti-rabbit IgG was used as the secondary antibody (1:2000, Bio-Rad Laboratories). Mouse anti-histone H3 antibody (1:10,000; Millipore, Billirica, MA, USA) was used as a loading control.

### CD133, cell cycle and cleaved caspase-3 assessment using flow cytometry

We combined CD133 measurement with cell cycle or with cleaved caspase-3 assessment for detection of apoptosis-sensitive cells in CD133− and CD133+ populations. UKF-NB-3 cells were cultured at a density of 2×10^5^ cells/ml and left for 24 hrs to adhere and then were treated with CDDP (1 μM) or VCR (0.20 nM) as well as in combination with 1mM VPA. After 72 hrs, the medium was removed and cells were rinsed twice with PBS. Attached cells were collected using accutase (Sigma-Aldrich), mixed gently in IMDM with 10% FBS and centrifuged. 10^6^ cells were re-suspended in 100 μL PBS containing 0.5% bovine serum albumin (Sigma-Aldrich) and then incubated with mouse anti-human CD133/2 PE-conjugated primary antibody clone 293C3 (1:10, Miltenyi Biotec) for 15 min in darkness at 4°C. Cells were washed and fixed with 3.6% paraformaldehyde/PBS for 10 min on ice. After washing, cells were permeabilized and nuclei stained with DAPI at a final concentration of 10 μg/ml (Life Technologies, CA, USA) in a 0.15% TritonX/PBS solution for 15 min on ice. Cells were washed and incubated with monoclonal anti-cleaved caspase-3 Alexa Fluor^®^647 conjugated antibodies clone D3E9 (1:50; Cell Signaling) for 30 min on ice. Labeled cells were measured immediately using LSR II Flow Cytometer (BD Bioscience, San Jose, CA, USA) and data were analyzed using FlowJo X software (Tree Star, Oregon, USA). Identically treated samples stained with Mouse IgG2b-PE antibodies (1:10; Miltenyi Biotec) were used as isotype controls. Effect of VPA on cell cycle using propidium iodide (PI) was assessed using DNA PREP Reagents kit (Beckman Coulter Inc., CA, USA) according to the manufacturer's instructions. The proliferation index was calculated using the following formula: proliferation index = (G_2_M + S)/ (G_0_G_1_ + S + G_2_M). All measurements were independently repeated at least three times. Figures show the result of one typical experiment.

### Annexin V/propidium iodide (PI) labeling

For detection of apoptosis, Annexin V-FITC Apoptosis Detection kit (Biovision, Milpitas, CA, USA) was used according to the manufacturer's instructions as we have previously described [42]. Samples were analyzed using LSR II flow cytometer (BD Bioscience). The sum values of early apoptotic cells (Annexin V+/PI-) and late apoptotic cells (Annexin V+/PI+) represented the total apoptosis.

### Immunofluorescence staining of CD133

UKF-NB-3 cells were fixed with 4% paraformaldehyde for 15 min at room temperature, washed three times with PBS, and then blocked with a PBS-based solution containing 1% bovine serum albumin and 0.25% Triton X-100 (Sigma-Aldrich). Cells were incubated overnight at 4°C with mouse monoclonal anti-CD133 (1:30, clone W6B3C1, Miltenyi Biotec). After being washed three times with PBS, cells were co-incubated with goat anti-mouse DyLight 488 IgG (1:400; Abcam). Nuclei were counterstained with DAPI (1μg/mL; Life Technologies). Immunofluorescent images were collected using Leica TCS SP5 confocal microscope (Leica Microsystems, Mannheim, Germany).

### Treatment with 5-aza-2’-deoxycytidine and promoter methylation profiling

Cells were cultured in a density of 4×10^5^ cells/ml and left to adhere overnight. Cultured cells were treated with 5-aza-2’-deoxycytidine (AZA) (Sigma-Aldrich) for 6 days at a dose of 4 μM for IMR-32 and 8 μM for UKF-NB-4. VPA was added 48 hrs before collecting the cells. The medium was changed and replaced with new AZA containing medium every other day. Adherent cells were collected for western blot and DNA isolation.

*Bisulfite conversion*: DNA was extracted using a Puregene Core KitA (Qiagen, Hilden, Netherlands). Whole genomic DNA was treated with sodium bisulfite using an Epitect Bisulfite Kit (Qiagen) to convert unmethylated cytosine to uracil, following the manufacturer´s protocol.

*Methylation-sensitive high resolution melting (MS-HRM)*: Real-time PCR followed by HRM was carried out using a high-performance Eco Real-Time PCR system (Illumina, San Diego CA, USA). CD133 primers specific for bisulfite converted DNA of the promoter P1 and P3 [43] were designed using Methyl Primer Express Software v1.0 (Applied Biosystems, Carlsbad, CA, USA) (S1 Table). The reaction mixture consisted of 10 ng of template DNA, 1x EpiTect HRM Master Mix (Qiagen) and 300 nmol/l of each primer. PCR was initiated by incubation at 95°C for 5 min, followed by 50 cycles at 95°C for 10 sec, 56°C for 20 sec, and 72°C for 10 sec. The HRM thermal profile was set up according to the manufacturer’s recommendations. For each assay, a standard dilution series using EpiTect Control DNA (Qiagen) was run. Fluorescence data were converted into melting peaks using Eco Software v3.0.16.0 (Illumina).

### MTT test

The IC_50_ of VPA, CDDP and VCR after 72 hrs of treatment in NB cell lines was assessed by MTT assay. The average value was calculated from at least 3 independent experiments using the linear regression of the dose-log response curves by SOFTmaxPro software as previously described [44]. For the dose response curve, a serial dilutions of mentioned drugs (0.1–50 mM VPA), (0.08–40 μM CDDP) and (0.04–20 nM VCR) were used. The absorbance at 570 nm was measured for each well by multiwell ELISA reader Versamax (Molecular Devices, Sunnyvale, CA, USA).

### Statistical analysis

Numerical data were expressed as mean ± standard deviation (SD). Paired Student’s t-test (two tailed) was used for statistical analysis using SPSS.v16.0 for windows (SPSS Inc., Chicago, IL, USA). *P* values less than 0.05 were considered statistically significant and indicated with *; *P* values less than 0.01 were indicated with **.

## Results

### Assessment of the cytotoxic effect of 1mM VPA on NB cell lines

Evaluation of the cytotoxic effect of 1mM VPA at 72 hrs was determined by IC_50_, apoptosis assessment using Annexin V-FITC/PI double staining assay and cell cycle assessment using PI. The average IC_50_ for UKF-NB-3, IMR-32, UKF-NB-4, and SH-SY5Y were 1.27, 1.3, 2, and 9 mM respectively (Table 1). These low IC_50_ values (except for SH-SY5Y) were mainly due to the inhibitory effect of VPA on cell cycle rather than apoptosis which did not exceed 5% at maximum in IMR-32 as compared to the control (Table 1). VPA reduced the cell number due to shifting of cells toward G_0_/G_1_ phases of the cell cycle which consequently decreased the proliferation index and resulted in remarkable difference in cell number between control cells and cells treated with VPA leading to low IC_50_ for MTT test (Table 1). Cell cycle inhibition associated with 1mM of VPA was markedly obvious in UKF-NB-3, IMR-32, and UKF-NB-4, while it was insignificant in SH-SY5Y during the examined period. Thus, the high IC_50_ value in SH-SY5Y represented mainly the apoptotic effect induced by VPA. Here, we would like to underscore that the VPA concentration we used in our experiments (1mM) is similar as recommended serum concentration of patients in clinical trials [45].

**Table 1:** IC_50_, apoptosis, and proliferation index of NB cell lines after treatment with 1mM VPA for 72 hrs.

Cell line	IC_50_VPA mM	Apoptosis Annexin V/PI labeling		Proliferation index (G_2_M + S) / (G_0_G_1_ + S + G_2_M)	
		Control	VPA	Control	VPA
**UKF-NB-3**	1.27 ± 0.46	2.27 ± 0.49	4.86 ± 1.74**	0.50 ± 0.05	0.43 ± 0.02*
**IMR-32**	1.30 ± 0.69	2.08 ± 0.26	7.02 ± 0.88**	0.55 ± 0.01	0.43 ± 0.01**
**UKF-NB-4**	2.00 ± 0.60	0.61 ± 0.15	4.72 ± 0.48**	0.64 ± 0.02	0.58 ± 0.01**
**SH-SY5Y**	9.00 ± 1.44	3.84 ± 1.02	7.38 ± 0.78**	0.35 ± 0.04	0.34 ± 0.03^Ns^

Ns, Non-significant

* *p* < 0.05

** *p* <0.01

- paired t-test

### VPA increased CD133 expression in association with increased acetylation of histone in NB cell lines

**Fig 1:**
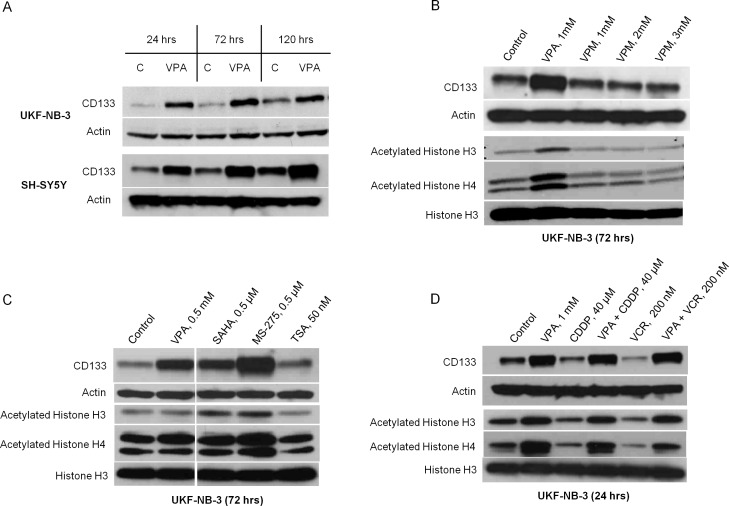
Changes of CD133 expression and histones H3 and H4 acetylation in UKF-NB-3 and SH-SY5Y after cultivation with 1mM VPA, valpromide, other HDAC inhibitors and cytostatics. (A) VPA increased CD133 expression in UKF-NB-3 and SH-SY5Y. (B) VPM failed to influence CD133 expression or histone acetylation after cultivation with non-toxic doses for 72 hrs. (C) CD133 expression correlated with the changes of histones H3 and H4 acetylation when cultured with different HDAC inhibitors. (D) CD133 expression correlated with the changes of histones H3 and H4 acetylation when cultured with high doses of CDDP or VCR and in combination with VPA for 24 hrs.

**Fig 2:**
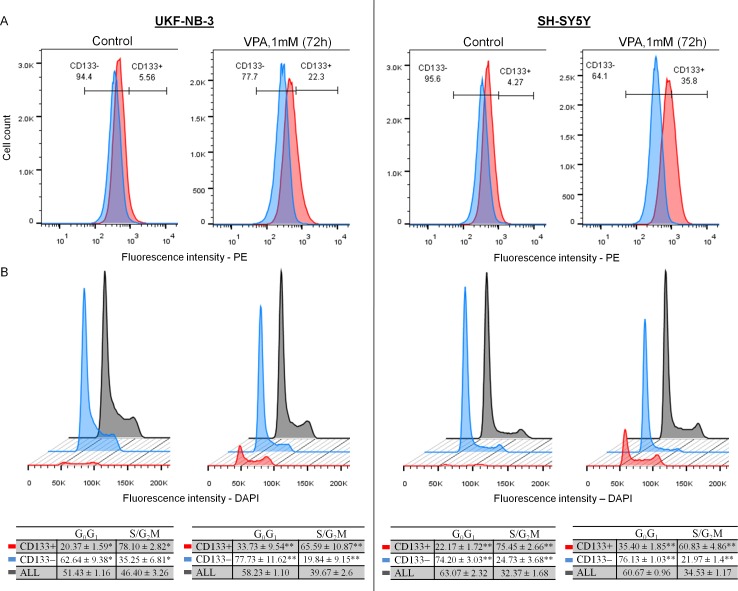
Cytometric assessment of CD133− and CD133+ and their cell cycle in control cells and after cultivation with 1mM VPA for 72 hrs in UKF-NB-3 and SH-SY5Y. (A) VPA Increased the number of CD133+ cells in both cell lines. *Blue*—isotype control (IgG2b-PE); *red*—CD133 expression detected by 293C3-PE (CD133/2) antibodies. (B) CD133+ cells were mainly located in the S and G_2_/M phases, while CD133− were mainly present in the G_0_/G_1_ phases.

VPA increased the expression of CD133 protein detected by western blot and the number of CD133+ cells detected by flow cytometry only in UKF-NB-3 and SH-SY5Y (Fig 1A and Fig 2A), while CD133 protein was not detected in UKF-NB-4 or in IMR-32 as revealed by western blot. The induction of CD133 protein by VPA was obvious during the first 24 hrs of cultivation and was gradually increasing over time (120 hrs). Noticeably, CD133 protein was consistently higher in VPA supplemented cultures than in control. Overexpression of CD133 protein following VPA treatment was accompanied by a rise of the CD133 mRNA as demonstrated in our previous article [46]. In addition, the small amount of apoptosis induced by 1mM VPA at 72 hrs excludes the possibility that CD133+ cells increased as a relative result of the reduction of CD133− cells. Treatment of the UKF-NB-3 cells with different concentrations of VPM (1mM, 2mM and 3mM) for 72 hrs showed no effect on the expression of CD133 as well as on the acetylation status of histones H3 and H4 at any of the tested concentrations (Fig 1B). These concentrations were not cytotoxic and did not induce cell cycle arrest (data not shown). Of note, VPM is a carboxamide derivative of VPA which is used as antiepileptic drug but it does not inhibit HDAC [47]. We also analyzed the expression of CD133 in UKF-NB-3 after 72 hrs incubation with different classes of HDAC inhibitors. Our results revealed that induction of CD133 by SAHA (0.5μM), MS-275 (0.5μM) and TSA (50nM) or a lower dose of VPA (0.5 mM) correlated with the changes in H3 and H4 acetylation. For example, the maximum acetylation we noticed was induced by entinostat and was associated with highest expression of CD133. Similarly, the low dose of TSA that we tested did not seem to affect the acetylation of histones H3 and H4 and was not associated with increased expression of CD133 (Fig 1C). Furthermore, treatment for 24 hrs with high doses of CDDP (40 μM, IC_50_/24 hrs = 11.12 μM ± 1.93) or VCR (200 nM, a dose that did not show cytotoxicity by MTT test within the first 24 hrs) was associated with low acetylation of histones H3 and H4 as well as a down-regulation of CD133 (Fig 1D). Thus, we noticed that changes of CD133 protein expression were accompanied by parallel changes in histones H3 and H4 acetylation level in all our experiments. Notably, we found that CD133 protein was not only located on the cell membrane but was also retained intracellularly as revealed by confocal microscope and cytometric measurements of intracellular CD133 expression. The intracellular positivity was increased significantly after treatment with 1mM VPA (S1 Fig and S2 Fig). This implies that CD133− cells detected by flow cytometry would express CD133 intracellularly after incubation with VPA.

### Re-expression of CD133 after treatment with 5-aza-2’-deoxycytidine

**Fig 3:**
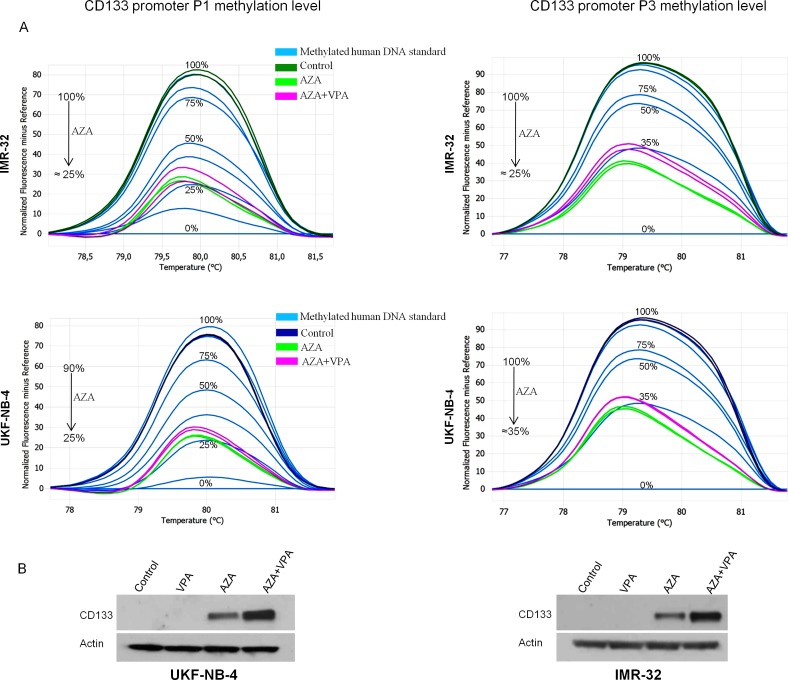
Normalized melt curves showing methylation status of promoters P1 and P3 in UKF-NB-4 and IMR-32 before and after using AZA and in combination with VPA. **Western blot is showing the effect of 1mM VPA and AZA on CD133 expression in methylated cell lines.** (A) Treatment with AZA decreased the methylation of both promoters P1 and P3 in UKF-NB-4 and IMR-32. (B) CD133 was not detected in cell lines with methylated promoters (UKF-NB-4, IMR-32). Re-expression of CD133 was in a small amount after applying AZA and in high amount when combined with VPA. VPA alone did not induce CD133 protein in UKF-NB-4 and IMR-32.

Methylation level of CD133 promoters P1 and P3 was assessed using MS-HRM analysis (S3 Fig). The methylation status of these promoters correlated with CD133 protein expression in all our cell lines. For instance, cell lines with highly methylated promoters (UKF-NB-4 and IMR-32) did not express CD133, while those with low methylation (UKF-NB-3 and SH-SY5Y) expressed CD133 as detected by western blot. To assess whether CD133 expression was silenced by methylation, cell lines that showed no expression of CD133, even after incubation with VPA (UKF-NB-4 and IMR-32), were treated with the demethylating agent AZA. We found that CD133 protein was restored again in a small amount after AZA treatment and in a larger amount when AZA was combined with VPA (Fig 3B). The re-expression of CD133 was associated with a drop in promoters P1 and P3 methylation levels (Fig 3A). The increase of CD133 expression after VPA did not seem to coincide with changes in CD133 promoter methylation.

### CD133+ NB cells were mainly located in the proliferative phases and were chemoresistant

We determined the chemoresistance of CD133+ and CD133− populations and their location along the cell cycle phases by co-staining cells with CD133 and cleaved caspase-3 or DAPI.

**Fig 4:**
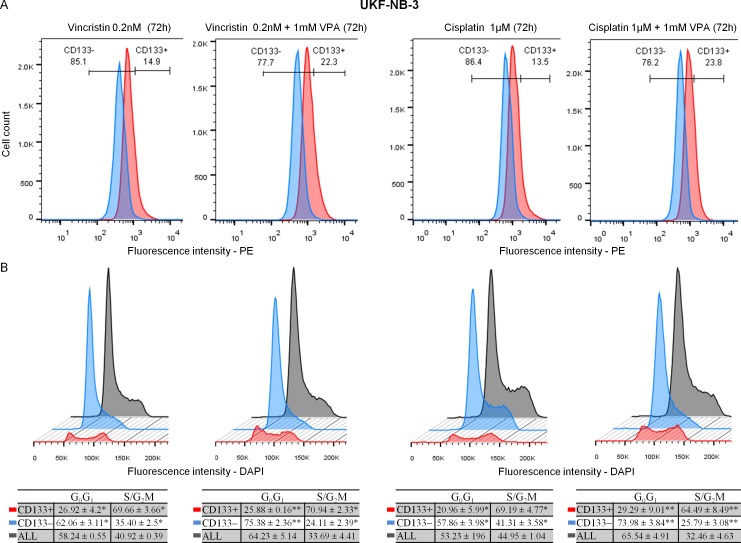
Changes of CD133 expression and cell cycle in VPA combined therapy at 72 hrs. (A) Increased CD133+ cells in VPA combined therapy. *Blue*—isotype control (IgG2b-PE); *red*—CD133 expression detected by 239C3 (CD133/2) antibodies. (B) CD133+ cells were mainly located in the S and G_2_/M phases, while CD133− were mainly present in the G_0_/G_1_ phases during treatment with cytostatics or in combination with VPA.

Our results showed that CD133+ cells either in UKF-NB-3 or SH-SY5Y were mainly located in the S and G_2_/M phases compared to CD133− cells which were predominantly seated in the G_0_/G_1_ phases whether in control or in samples incubated with 1mM VPA for 72 hrs (Fig 2B). This indicates that CD133+ cells represent a proliferating fraction even under the cell cycle inhibitory effect of VPA. CD133+ cells were also induced in VPA combined therapy (Fig 4A) and were predominantly located in the S/G_2_M phases during treatment with 1 μM CDDP or 0.20 nM VCR and in combination with 1mM VPA for 72 hrs (Fig 4B).

**Fig 5:**
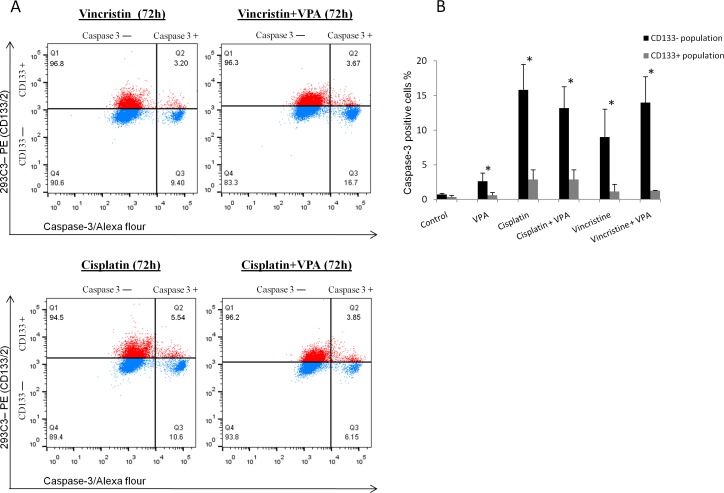
Activated caspase-3 in CD133– and CD133+ populations after treatment with VPA combined therapy for 72 hrs. (A) Activated caspase-3 was higher in CD133− cells compared to CD133+ cells. *Blue*—isotype control (IgG2b-PE); *red*—CD133 expression detected by 239C3 (CD133/2) antibodies. (b) Mean cleaved caspase-3 was significantly higher in CD133− than in CD133+ cells in all tested samples.*p < 0.05—paired t-test.

We detected the cleaved caspase-3 in CD133+ and CD133− populations in UKF-NB-3 cell line after incubation with 1 μM CDDP (IC_50_ /72 hrs = 0.97 μM ± 0.10), 0.20 nM VCR (IC_50_ /72 hrs = 0.22 nM ± 0.03) and in combination with 1mM VPA for 72 hrs. The percentage of cleaved caspase-3 was significantly higher in CD133− compared to CD133+ cells whether using cytostatics alone or in combination with VPA (Fig 5A and 5B).

### VPA pretreated samples acquired higher colony and neurosphere formation capacity in UKF-NB-3

**Fig 6:**
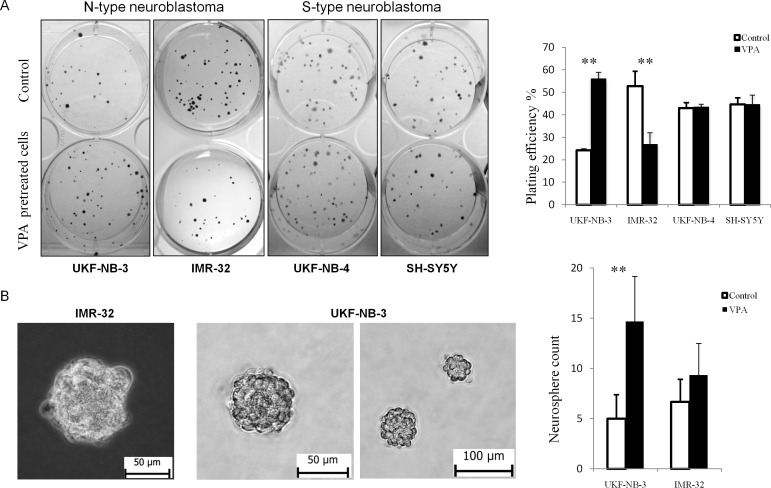
Effect of 1mM VPA on clonogenicity and neurosphere formation in NB cell lines. (A) Pretreatment with VPA significantly increased the clonogenicity in UKF-NB-3, while it decreased in IMR-32. In S-type NB (UKF-NB-4, SH-SY5Y) clonogenicity was not affected by pretreatment with VPA. VPA- cells pretreated with 1mM VPA for 72 hrs. (B) Both UKF-NB-3 and IMR-32 were able to form neurospheres in SFM. Pretreatment with 1mM VPA increased the number of formed neurospheres with high statistical significance in UKF-NB-3.

UKF-NB-3 cells pretreated with VPA showed significantly higher colony forming capacity compared to control, while the IMR-32 cell line which lacks for CD133 had fewer colonies than control (Fig 6A). There was no significant difference in the clonogenicity either in SH-SY5Y [48] or UKF-NB-4 when pretreated with VPA.

Since sphere forming capacity is an indicator of the self-renewal activity of CSCs *in vitro*, we examined the effect of 72 hrs pretreatment with 1mM VPA on the frequency of neurospheres formation. UKF-NB-4 and SH-SY5Y cells maintained in SFM were strictly adherent to the culture floor forming monolayer spreading without neurosphere formation. UKF-NB-3 and IMR-32 grown in SFM consistently formed organized neurospheres. UKF-NB-3 cells pretreated with VPA showed significantly higher capacity for neurospheres formation than control cells. IMR-32 pretreated with VPA showed slight increase in the neurospheres formation that was statistically insignificant (Fig 6B).

### NB cell lines with high CD133 protein content expressed higher phosphorylated Akt

**Fig 7:**
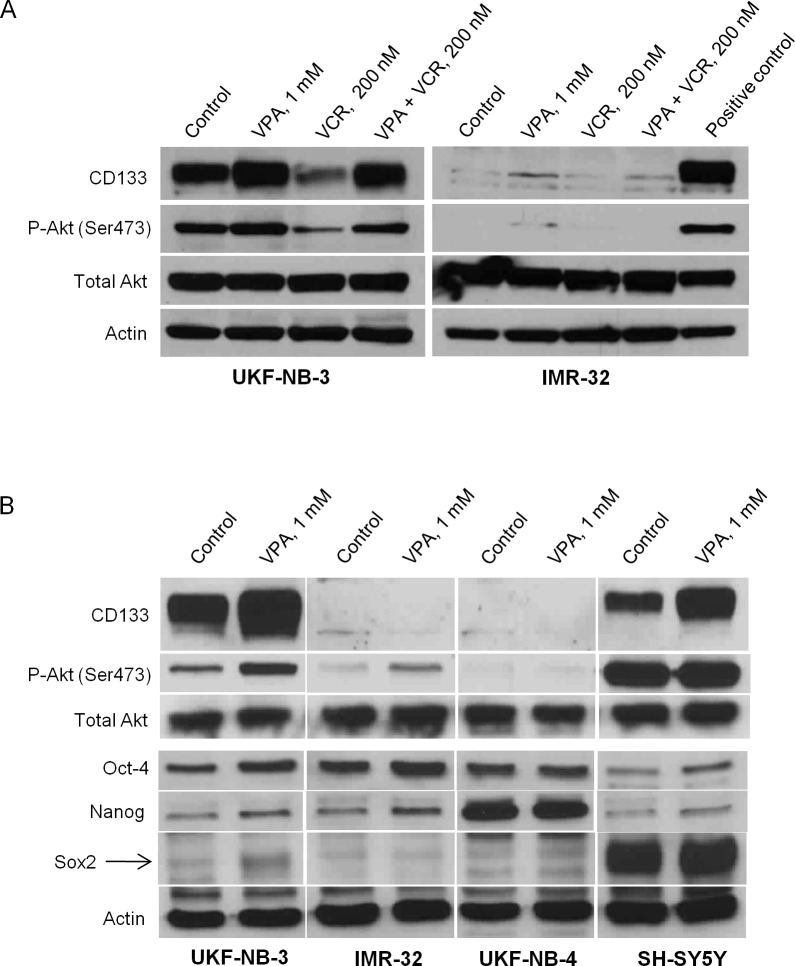
Expression of CD133, P-Akt, Oct-4, Nanog and Sox2 in NB cell lines after treatment with 1mM VPA. (A) Changes of P-Akt in UKF-NB-3 were correlated to the changes of CD133 under all examined conditions (1mM VPA or 200nM VCR for 24 hrs). Positive control- UKF-NB-3 cells treated with 1mM VPA for 24 hrs. (B) 72 hrs cultivation with VPA induced CD133 overexpression only in UKF-NB-3 and SH-SY5Y cell lines. P-Akt was highly expressed in CD133-expressing cell lines. Phophorylation of Akt increased with VPA treatment in UKF-NB-3, whereas its expression was extremely high in SH-SY5Y whether in the control or VPA treated sample. Oct-4 was mainly expressed in MYCN amplified cell lines (UKF-NB-3, IMR-32 and UKF-NB-4) and was enhanced after treatment with VPA in UKF-NB-3, IMR-32 and SH-SY5Y. Nanog expression was detected in low amount that enhanced by VPA in UKF-NB-3, IMR-32 and SH-SY5Y, while it was highly expressed in UKF-NB-4. SOX2 was slightly expressed in UKF-NB-3 and was induced by VPA while it was over expressed in SH-SY5Y independent of VPA effect.

We compared the P-Akt (Ser 473) in two N-type NB cell lines, UKF-NB-3 which expressed high level of CD133 protein and IMR-32 which contained highly methylated CD133 promoters and was not expressing CD133 protein. The association of CD133 and Akt activation was tested through detection of P-Akt in different conditions that markedly influence the CD133 expression such as treatment with 1mM of VPA, with high dose of VCR (200mM) and their combination for 24 hrs. Our results clearly demonstrated a higher basal level of P-Akt in UKF-NB-3 than in IMR-32 where neither the CD133 nor the P-Akt was detected. The changes of P-Akt in UKF-NB-3 correlated with the changes of CD133 under all examined conditions (Fig 7A). Treatment with 1 mM VPA for 72 hrs enhanced the expression of P-Akt in UKF-NB-3 significantly while a mild increase of P-Akt in IMR-32 was noticed. The level of P-Akt in SH-SY5Y was extremely high whether in control or in cells treated with VPA. P-Akt was not detected in UKF-NB-4 under the tested condition.

### Expression of pluripotent factor Oct-4 is a feature of MYCN amplified NBs and can be enhanced by VPA treatment

We have examined the expression of stem cell transcription factors Oct-4, Sox2 and Nanog in NB cell lines by western blot. We found that Oct-4 is highly expressed in MYCN amplified cell lines UKF-NB-3, UKF-NB-4 [49] and IMR-32 [50] and to a lesser extent in MYCN non-amplified cell line SH-SY5Y [50]. Treatment with VPA obviously enhanced the protein expression of Oct-4 in UKF-NB-3, IMR-32 and SH-SY5Y (Fig 7B). On the other hand, Sox2 has been extensively expressed in SH-SY5Y and its low expression in UKF-NB-3 was enhanced by VPA treatment. Nanog was detected in low amount in UKF-NB-3, IMR-32 and SH-SY5Y that was enhanced by VPA treatment while it was overexpressed in UKF-NB-4 either in the control or VPA treated samples (Fig 7B). Collectively, VPA enhanced the expression of stemness related markers (Oct-4, Sox2 and Nanog) in variable amounts in NB cell lines particularly in those expressing CD133 (UKF-NB-3 and SH-SY5Y).

## Discussion

CD133 is a complex gene in its structure with seven splice variants and its protein expression pattern is still debated. Several authors have shown that sorted CD133+ cells displayed greater tumorigenicity and resistance to chemotherapeutic agents compared to CD133– cells. However, other researchers demonstrated that CD133– cells can display similar capacities as CD133+ [51,52]. The reason for this contradiction can be explained by some factors which regulate and alter CD133 expression such as the glycosylation pattern [53], the change in the bioenergetic status (hypoxia and mitochondrial dysfunction) [54] and more importantly is the conclusion that CD133 protein expression is not synonymous to cell surface epitopes immunoreactivity [53,55].

In our study, we found that CD133 molecule can be located intracellularly as well as on the cell membrane. Treatment with VPA increases the intracellular expression of CD133 almost in all cells. For this reason we performed the cytometric staining of CD133 before permeabilization of cell membrane to ensure that the detected CD133+ cells are those expressing CD133 epitope on cell membrane and not intracellularly. This remark is of great value when working with CD133 to ensure the precise detection of CD133+ cells.

In our article, we examined the epigenetic effects of the HDAC inhibitor VPA on the expression of CD133+ in NB and we gave overview about the variability of CD133 protein expression in relation to the acetylation of histones and methylation status of CD133 promoters.

We used two antibodies that can identify different epitopes of the CD133 protein, W6B3C1 (CD133/1) that we used for immunoblotting and 293C3 (CD133/2) for flow cytometric analysis. Even though 293C3 (CD133/2) antibody recognizes different epitope than AC133 (CD133/1) (the conventional antibody used to detect and isolate CSCs), it has shown similar sensitivity in detecting CD133+ cells [53]. Our results offer evidence for induction and maintenance of CD133+ cells in NB cell lines through inhibition of HDAC by VPA. We relate this effect to its action on increasing the acetylation of histones which leads to chromatin accessibility and consequent transcription of many genes including CD133. In contrast, valpromide (carboxamide derivative of VPA that does not inhibit HDAC) did not alter the histone acetylation and was not accompanied by any change in the expression of CD133. We noticed a parallel interaction between CD133 expression and the acetylation of histones H3 and H4 in all our experiments. A lower dose of VPA that was used in clinical trials [45] as well as different classes of HDAC inhibitors increased the expression of CD133 in variable degrees that were correlated positively with the amount of H3 and H4 acetylation. This suggests the concomitant relation between CD133 expression and acetylation of histones H3 and H4 induced by HDAC inhibitors.

Interestingly, we noticed down-regulation of CD133 when treated with high doses of VCR and CDDP, which should not be interpreted to mean that CD133 permanently disappeared, because the same cells were able to significantly express CD133 when combined with VPA. This variability in CD133 expression is under control of the epigenetic modification which could explain why sorted CD133− cells can acquire the CD133 surface marker or CD133+ cells can lose it in some tumors [56].

We also showed that CD133 could be controlled by methylation of CpG promoters that can silence the expression of CD133 protein. In our study, CD133 was dramatically increased after using VPA in cell lines with unmethylated P1 and/or P3 promoters (UKF-NB-3 and SH-SY5Y). On the other hand, cell lines with methylated P1 and/or P3 promoters (UKF-NB-4 and IMR-32) failed to show any increase in CD133 after VPA treatment. CD133 expression in those lines was only restored by demethylating agent AZA and can be enhanced if the demethylation is followed by increasing histone acetylation by VPA. Thus, our work is in agreement with Shmelkov et al., who stated that promoter methylation may play a key role in CD133 regulation [43].

In our study, VPA induced CD133 protein which was reflected on the increase of CD133+ cells as detected by flow cytometry. We mentioned in our previous results that the CD133+ cells induced by VPA treatment was preceded by increase in the mRNA [46]. The point to highlight here is that VPA had the potential to turn cells that were detected as CD133− using flow cytometry into CD133+. This led us to suggest two possibilities: first, VPA could maintain the CD133+ cells undifferentiated which explain the consistent expression of surface CD133 epitopes in VPA treated samples. Second, CD133− cells were able to synthesize CD133 protein and express it in accessible pattern on cell surface, thus acquire the characters of CD133+ cells. This action of VPA can be related to the effect of HDAC inhibitors in cell reprogramming and induction of pluripotent cells [9].

In fact, we detected variable expression of pluripotent transcriptional factors Oct-4, Sox2 and Nanog in CD133 expressing cell lines and they were obviously induced by VPA treatment. Although Oct-4 and Nanog were also expressed in NBs lines with MYCN amplification such as IMR-32 and UKF-NB-4, these cell lines did not show evidence of Sox2 protein expression which was only detected in CD133 expressing cell lines. A previous reports have demonstrated that Oct-4 and Sox2 orchestrate together to maintain the self-renewal and pluripotency of embryonic and neural stem cells [57]. However, Oct-4 is considered as a master pluripotency gene and is expressed in side-population cells of NB [58]. Knockdown of Oct-4 inhibited the formation of spheres and the CD133 expression in MYCN-amplified human NBs cells [59]. Additionally, high Oct-4 expression was correlated with poor prognosis especially in patients with MYCN-amplified NBs [59]. Based on previous remarks, combined induction of these transcription factors together with CD133 expression after VPA treatment in MYCN amplified cell line (UKF-NB-3) might suggest induction of pluripotency. According to previously mentioned results, it was necessary to perform further experiments to assess the self-renewal and chemoresistance of VPA treated UKF-NB-3 cells.

Human NB cells occur in three different subtypes, S-type, N-type, and I-type, each with distinct morphology and behavior. S-type cells resemble glial precursor cells, which are highly substrate adherent, and are non-invasive. N-type cells have a neuronal morphology, and are less substrate adherent, but highly invasive [60]. In our study, pretreatment with VPA significantly enhanced colony formation capacity in the N-type NB cell line UKF-NB-3 but not IMR-32, which lacks the CD133 protein. There was no difference in colony formation regarding the S-type NB. We suggest that pretreatment with a low dose of VPA can enhance colony formation capacity of susceptible tumors, which may be related to the increase in CD133+ cells. Additionally, it has been reported the role of CD133 in cell adhesion [29] which greatly can affect the colony formation in N-type with low attachment characters rather than the S-type with very high attachment ability. Therefore, we examined the capacity of neurospheres formation which can be more reliable test for the self-renewal activity *in vitro*. We found that VPA significantly increased the number of formed neurospheres in UKF-NB-3 but not in IMR-32, whereas the S-type NBs consistently formed adherent colonies.

We used the cytometry to examine the cell cycle and apoptosis-sensitive cells within the pool of the cultured cells. We demonstrated that CD133+ cells are mainly present in the S/G_2_M phases, while CD133− cells largely reside in G_0_/G_1_ either in the control or in samples treated with VPA or cytostatics which agrees with other published results [55]. Stem cells are generally dormant *in vivo*, they tend to proliferate to regenerate tissue loss [61]. Similarly, cell lines growing in exponential phase seem to be a model of this regeneration [25]. In particular, evidence suggests that CSCs may arise from normal stem cells, progenitor cells or more differentiated cells with dysregulated proliferation [62] that matches with our findings and emphasis that CD133+ cells form a characteristic population in tumor. Previous results clarified that CD133 mRNA knockdown in highly expressing CD133 NB cells effectively resulted in significant growth retardation in adherent cell cultures [16]. Moreover, CD133 was considered as a marker of specific stages of the cell cycle (S, G2 or M) in CSCs [63]. Altogether, we suggest that CD133+ cells can represent a significant proliferative fraction in NB cell lines.

Caspase-3 is the effector caspase that cleaves some cellular proteins including caspase-activated DNAase, which causes DNA fragmentation that is characteristic for apoptosis [64]. Therefore, low level of cleaved caspase-3 detected in CD133+ compared to CD133− cells in UKF-NB-3 cell line when treated with VPA and cytostatics, indicates resistance to apoptosis. This is in agreement with published result showing lower sensitivity of sorted CD133+ NB cells to cisplatin, carboplatin, etoposide, and doxorubicin compared to CD133− cells [28]. Interestingly, we detected a high level of P-Akt (active form) in UKF-NB-3 and SH-SY5Y in contrast to IMR-32 which lacks for CD133 protein and showed low level of activated Akt. The level of activated Akt in UKF-NB-3 was enhanced by VPA therapy and repressed by high dose of VCR that goes along with the changes in the CD133 expression in both conditions. The level of P-Akt in SH-SY5Y was extremely high and did not show higher expression when treated with VPA. Our results is supported by previous studies reported the association between the expression of CD133 in different cancers and activation of Akt pathway [32,65]. The activation of Akt is proposed to trigger pro-survival signaling via Akt mediated cell cycle arrest and anti-apoptotic mechanisms leading to chemotherapeutic resistance [66]. Altogether, we supposed that cancer cells expressing CD133 may get benefits from activation of Akt signaling during VPA therapy such as resisting apoptosis which manifested by low activated caspase-3 in our study.

## Conclusion

In conclusion, we found that CD133 expression in NB can be regulated by histones acetylation and/or methylation of its CpG promoters. Thus, we showed that the HDAC inhibitors can increase CD133 significantly in NB cell lines that show low methylated CpG promoters. We also found that VPA treatment may increase CD133+ cells that show higher resistance to cytostatics than CD133– cells. VPA treatment enhanced the ability of CD133 expressing cell line UKF-NB-3 to generate more colonies and neurospheres, induce Akt phosphorylation, induce expression of the pluripotency transcriptional factors which collectively may lead to induce chemoresistance and preserve tumor growth. Amplification of cancer stem like cells is unwanted action of VPA which antagonizes the idea of cancer stem cell theory that gives attention to target the CSCs rather than the more differentiated tumor cells. No doubt that addition of high doses of VPA to conventional chemotherapy has revealed a synergistic effect on cell cycle inhibition and apoptosis induction in tumor cells but we have to keep in mind that VPA dose is limited by its side effects and has different anti cancer effect depending on tumor cell biology. Therefore, it is of great value to determine the type of tumors that can be beneficial from VPA therapy. Finally, further investigations are needed for evaluation of epigenetic therapy (HDAC inhibitors and demethylating agents) on induction of CSCs in tumors prior to use in clinical medicine.

## Supporting Information

S1 Fig Intracellular immunostaining of CD133 after treatment with 1 mM VPA for 72hrs. (TIF)

S2 Fig Comparison of the cytometric measurements of the surface and intracellular staining of CD133 in UKF-NB-3. CD133 was present on the surface as well as intracellularly either in the control or after treatment with VPA. VPA increased CD133 surface expression significantly and enriched almost all cells with CD133 molecule intracellularly. Intracellular staining was performed through fixation and permeabilization of cells in two steps using paraformaldehyde 3.6% followed by a 0.15% TritonX / PBS. Data were analyzed using Flowlogic software (Inivai Technologies, Mentone, Australia).
(TIF)

S3 Fig Normalized melt curves showing methylation status of promoters P1 and P3 in examined cell lines. In UKF-NB-3, methylation of both promoters were from 0–10%, in SH-SY5Y (P1 = > 90%, P3 = 0%), in UKF-NB-4 (P1 = 90%, P3 = 100%), IMR-32(P1 = 100%, P3 = 100%).
(TIF)

S1 Table CD133 primers specific for bisulfite converted DNA of the promoter P1 and P3. (DOCX)

## Abbreviations

AZA: 5-aza-2’-deoxycytidine
bFGF: basic fibroblast growth factor
CDDP: Cisplatin
CSC: cancer stem cell
DAPI: 4',6-Diamidino-2-Phenylindole
DMSO: Dimethyl sulfoxide
EGF: Epidermal growth factor
FACS: Fluorescence-activated cell sorting
FBS: Fetal bovine serum
FDA: Food and drug administration
HDAC: Histone deacetylase
Hrs: Hours
IC_50_: Half maximal inhibitory concentration
IMDM: Iscove’s modified Dulbecco’s medium
MS-2753: Entinostat
MS-HRM: Methylation-sensitive high resolution melting
NB: Neuroblastoma
PBS: Phosphate-buffered solution
PE: Phycoerythrin
PI: Propidium iodide
SAHA: Suberoylanilide hydroxamic acid
SFM: Serum free medium
TSA: Trichostatin A
VCR: Vincristin
VPA: Valproic acid
VPM: Valpromide
