# Supplementary figures and images for: Valproic Acid Increases CD133 Positive Cells that Show Low Sensitivity to Cytostatics in Neuroblastoma
Source: PLoS One. 2016 Sep 14;11(9):e0162916. doi: 10.1371/journal.pone.0162916 (PMC5023141; doi:10.1371/journal.pone.0162916)

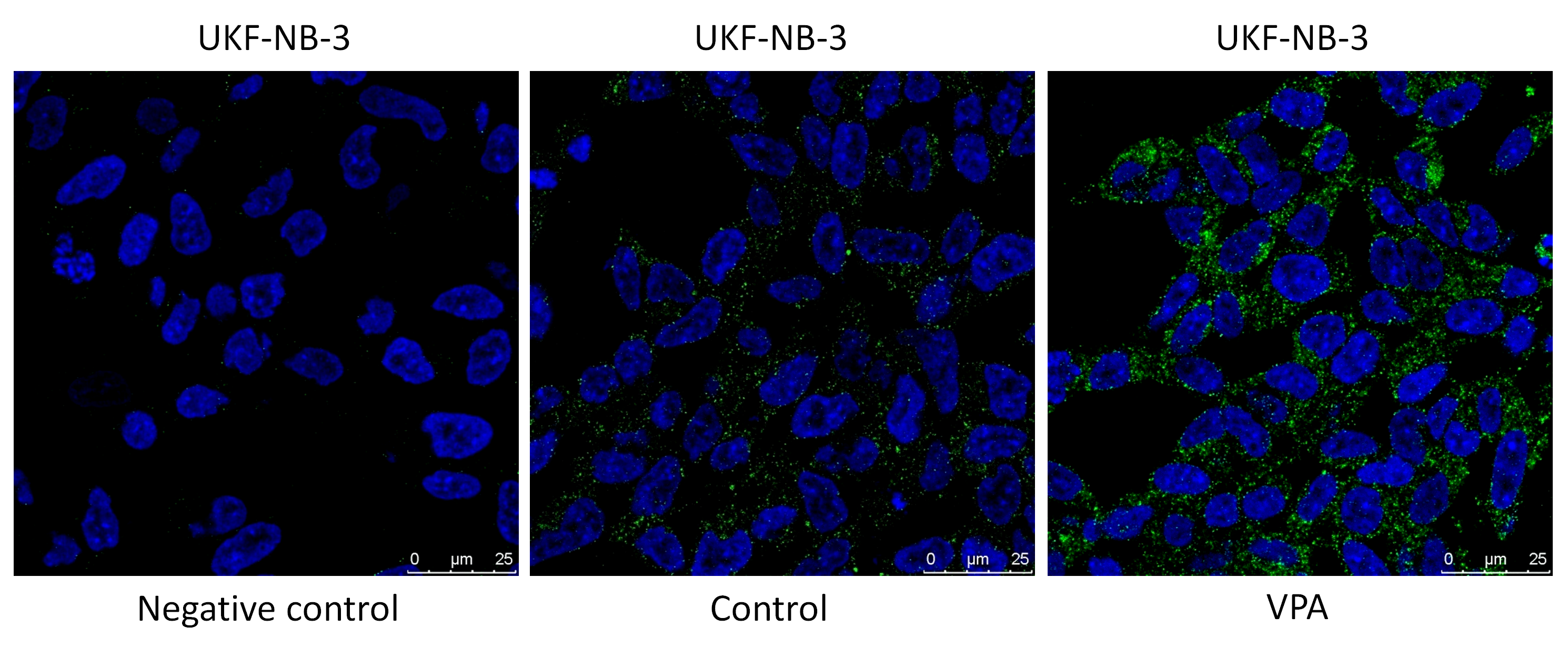

Supplement: S1 Fig — (TIF) [file pone.0162916.s001.tif]

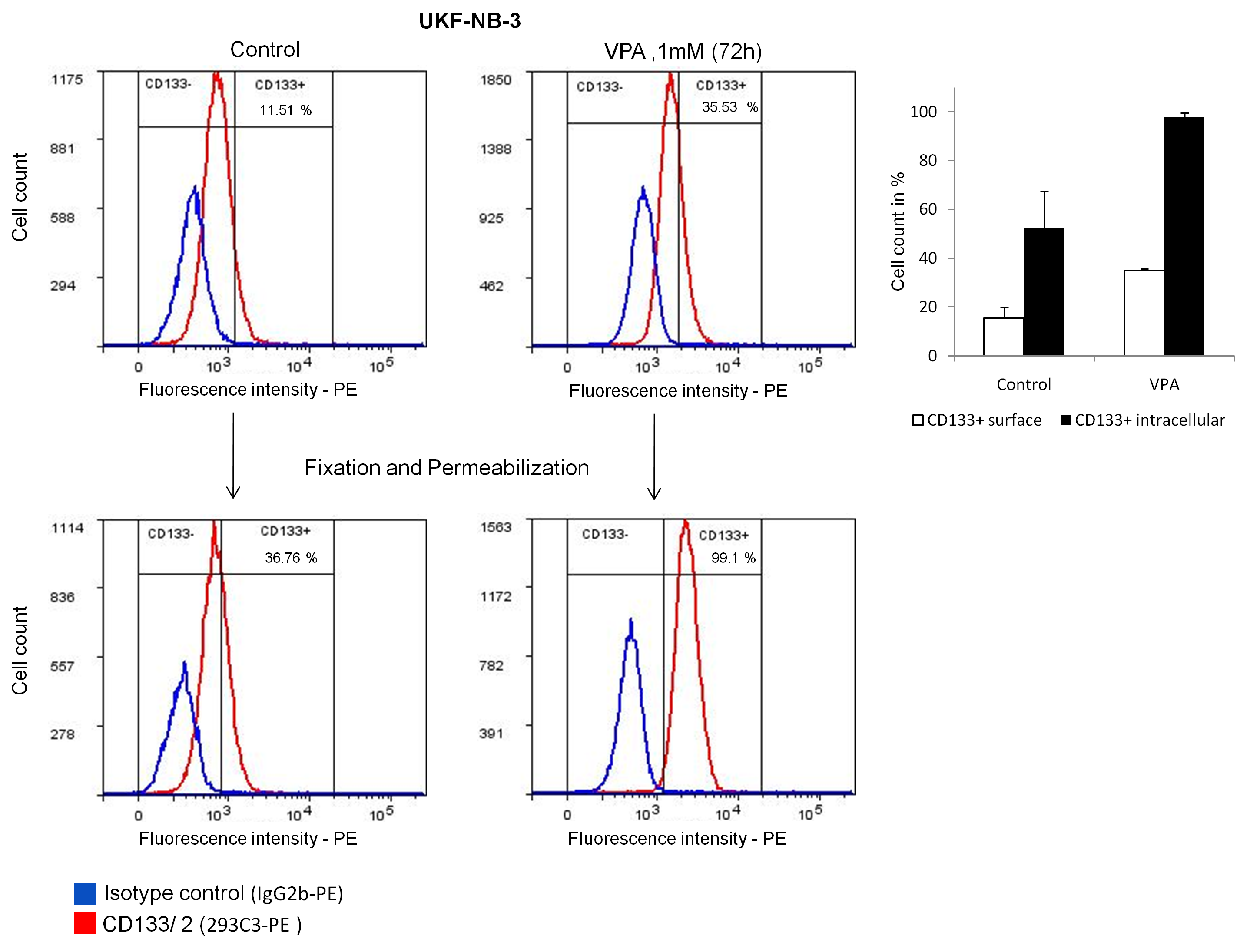

Supplement: S2 Fig — CD133 was present on the surface as well as intracellularly either in the control or after treatment with VPA. VPA increased CD133 surface expression significantly and enriched almost all cells with CD133 molecule intracellularly. Intracellular staining was performed through fixation and permeabilization of cells in two steps using paraformaldehyde 3.6% followed by a 0.15% TritonX / PBS. Data were analyzed using Flowlogic software (Inivai Technologies, Mentone, Australia). (TIF) [file pone.0162916.s002.tif]

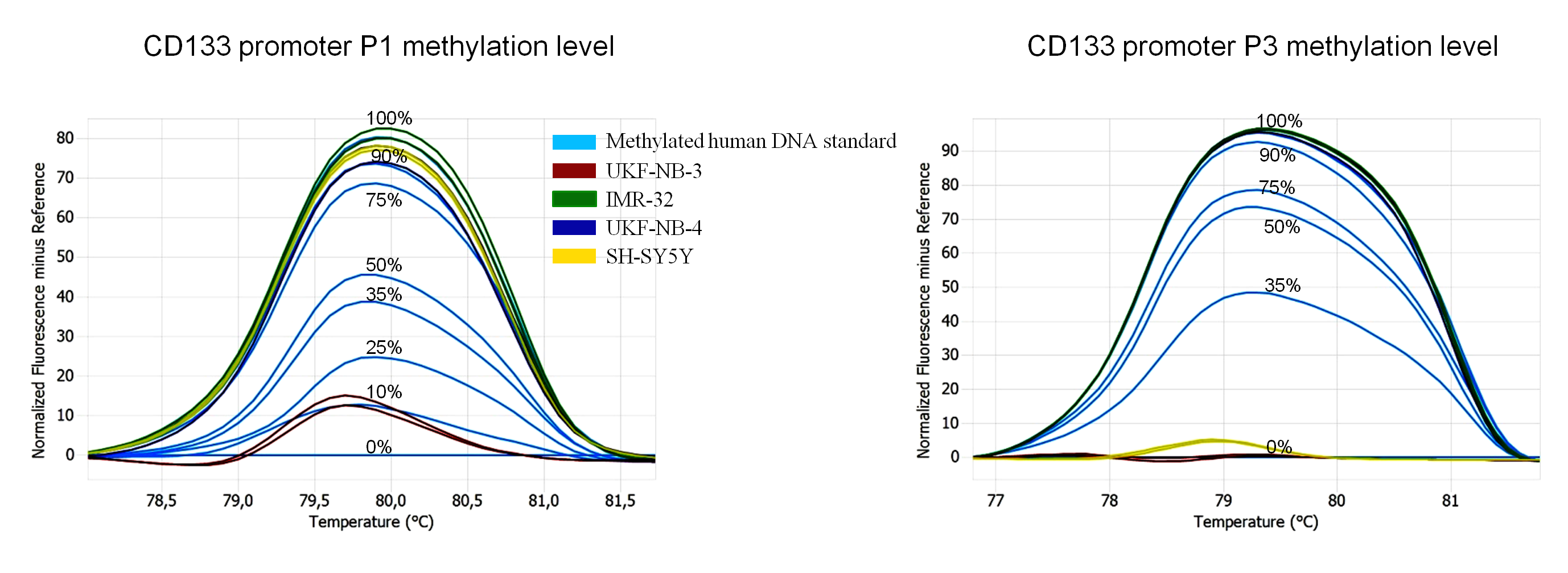

Supplement: S3 Fig — In UKF-NB-3, methylation of both promoters were from 0–10%, in SH-SY5Y (P1 = > 90%, P3 = 0%), in UKF-NB-4 (P1 = 90%, P3 = 100%), IMR-32(P1 = 100%, P3 = 100%). (TIF) [file pone.0162916.s003.tif]
