# Supplementary material for: Valproic Acid Increases CD133 Positive Cells that Show Low Sensitivity to Cytostatics in Neuroblastoma
Source: PLoS One. 2016 Sep 14;11(9):e0162916. doi: 10.1371/journal.pone.0162916 (PMC5023141; doi:10.1371/journal.pone.0162916)
Supplement: S1 Table — (DOCX) [file pone.0162916.s004.docx]

**S1 Table.**

| Primer name | Primer sequence | Tm [°C] | Ta [°C] | [bp] | Number of CpG |
| --- | --- | --- | --- | --- | --- |
| HRM-P1-F | 5' TGGGATTAGGTAATAGAAGGGTT 3' | 60.5 | 56 | 179 | 5 |
| HRM-P1-R | 5' CAACACCTAAACAACATCCATT 3' | 60.2 |  |  |  |
| HRM-P3-F | 5' TTATTGTATTGGGGGTGTATAGTGA 3' | 61.9 | 58 | 135 | 8 |
| HRM-P3-R | 5' CAATTCCTCTAACCCCCAAC 3' | 62.1 |  |  |  |

[bp], base pairs of the PCR product;[Ta], annealing temperature of primers; [Tm], melting temperature of primers.
